# Mitochondrial Dysfunction as a Biomarker of Illness State in Bipolar Disorder: A Critical Review

**DOI:** 10.3390/brainsci14121199

**Published:** 2024-11-28

**Authors:** Anna Giménez-Palomo, Helena Andreu, Oscar de Juan, Luis Olivier, Iñaki Ochandiano, Lidia Ilzarbe, Marc Valentí, Aldo Stoppa, Cristian-Daniel Llach, Giulio Pacenza, Ana Cristina Andreazza, Michael Berk, Eduard Vieta, Isabella Pacchiarotti

**Affiliations:** 1 Bipolar and Depressive Disorders Unit, Hospìtal Clinic de Barcelona, c. Villarroel, 170, 08036 Barcelona, Spainhandreug@clinic.cat (H.A.); ojviladegut@clinic.cat (O.d.J.); astoppa@clinic.cat (A.S.);; 2 Institut d’Investigacions Biomèdiques August Pi i Sunyer (IDIBAPS), c. Villarroel, 170, 08036 Barcelona, Spain; 3 Institute of Neurosciences (UBNeuro), 170 Villarroel St., 08036 Barcelona, Spain; 4 Mood Disorders Psychopharmacology Unit, University Health Network, Toronto, ON M5G 1M9, Canada; cristian-daniel.llachlopez@uhn.ca; 5 Department of Psychiatry, University of Toronto, Toronto, ON M5S 1A8, Canada; 6 Department of Pharmacology and Toxicology, University of Toronto, Toronto, ON M5S 1A8, Canada; 7 Mitochondrial Innovation Initiative, MITO2i, Toronto, ON M5S 1A8, Canada; 8 The Institute for Mental and Physical Health and Clinical Translation (IMPACT), School of Medicine and Barwon Health, Deakin University, Geelong, VIC 3220, Australia; 9 Departament de Medicina, Facultat de Medicina i Ciències de la Salut, Universitat de Barcelona (UB), c. Casanova, 143, 08036 Barcelona, Spain

**Keywords:** bipolar disorder, mitochondrial function, mental health, mood disorders

## Abstract

Mitochondria are organelles involved in different cellular functions, especially energy production. A relationship between mitochondrial dysfunction and mood disorders, especially bipolar disorder (BD), has been reported in the scientific literature, which suggests altered energy production and higher levels of oxidative stress compared to healthy controls. Specifically, in BD, the hypothesis of a biphasic pattern of energy availability has been postulated according to mood states. Current evidence highlights the presence of mitochondrial dysfunction in BD and variations between the manic, depressive, and euthymic phases. These findings need to be confirmed in future studies to identify biomarkers that may lead to individualized management of patients with BD and also to identify profiles with a higher risk of presenting an unfavorable course of illness, which would enable the design of preventive and therapeutic strategies in determined subpopulations of patients with BD. The limitations of this review include the non-systematic methodology, variety of mitochondrial-related functions associated with BD, heterogeneous study designs, preliminary evidence for specific findings, and limited recommendations regarding the use of mitochondrial modulators in BD.

## 1. Bipolar Disorder

Bipolar disorder (BD) is a chronic psychiatric disorder characterized by episodes of depression, mania, hypomania, and mixed states, which are interspersed with periods of clinical remission or euthymia [1,2]. Even with appropriate treatment, BD often adversely affects patients’ quality of life and psychosocial functioning, particularly in those with depressive symptoms, a higher number of past episodes, longer illness duration, and lower cognitive function [3].

This disorder has an estimated prevalence of 0.6% for BD type I, 0.4% for BD type II, and 2.4% for the broader bipolar spectrum [2,3]. The disorder typically emerges during late adolescence or early adulthood [3]. Early onset often correlates with delays in diagnosis and treatment. BD type II is more commonly seen in females, while BD type I affects males and females at similar rates [4]. Overall, BD has a heritability rate of approximately 60%, which is higher than that of many other mental disorders. It is a polygenic condition with substantial genetic overlap with other mental illnesses, such as major depressive disorder and schizophrenia, with more than 100 loci identified from genome-wide association studies (GWAS) shared by both BD and schizophrenia [2,5,6]. Despite the high heritability of BD, environmental factors, such as childhood adverse life events, drug misuse, and perinatal events, can modify the onset and course of this illness [7,8]. Specific treatments, such as antidepressants and corticosteroids, and medical conditions, including multiple sclerosis, stroke, autoimmune disorders, and endocrine disorders, have also been associated with BD onset. Finally, changes in seasons and light exposure have been described as triggers and course modifiers of BD [2].

Individuals with BD have a reduced life expectancy of nearly 13 years compared to the general population, with mortality rates two to three times higher [9]. The increased mortality rates are due to both natural causes, such as cardiovascular diseases, respiratory diseases, and cancer, and an elevated risk of unnatural deaths, particularly suicide [9].

Despite advances in neurobiological research, the pathophysiology of BD remains unclear. The different roles of mitochondria, including energy production, mitochondrial dysfunction, calcium homeostasis, apoptosis, and impaired energy metabolism, have been proposed as mechanisms involved in the development, severity, and progression of BD [10,11,12,13].

Growing evidence supports the hypothesis that BD is triggered, in part, via mitochondrial dysfunction, which is also associated with treatment outcomes and disease progression and severity [14,15], and that a biphasic pattern of energy availability depending on mood state might be found in this population [11], with increased mitochondrial activity in manic states and decreased function in depressive episodes. Altered mitochondrial dynamics, described in individuals with BD, also support the role of mitochondrial dysfunction in the course of the illness. These findings highlight the critical importance of obtaining new evidence in this field to better understand the underlying pathophysiology of this condition, which could facilitate the development of targeted interventions aimed at restoring energy homeostasis and mitigating mood fluctuations, and the identification of biomarkers for disease progression, treatment response, and relapse prediction, paving the way for personalized therapeutic strategies to improve long-term outcomes in BD. This article aims to review the available evidence regarding the implications of mitochondrial dysfunction in the pathophysiology of BD and explore novel targeted therapies acting on mitochondrial pathways that might be used in the treatment of BD. To this end, a literature search was conducted in PubMed and Cochrane Library in 2024 to identify available evidence regarding the role of mitochondrial function in the different phases of BD.

## 2. Mitochondrial Function

### 2.1. Mitochondria: Structure and Functions

Mitochondria are cellular organelles critical for numerous biological processes. They are described as the powerhouse of the cell and play essential roles in maintaining metabolic homeostasis through their involvement in energy production, reactive oxygen species (ROS) metabolism, calcium homeostasis, regulation of apoptotic cell death, synaptic plasticity, and neurogenesis, thereby modulating neuronal activity and preventing neuronal damage [16]. Mitochondria exist in almost all eukaryotic cells. According to the endosymbiotic theory, mitochondria originated from two prokaryotes that formed a symbiotic relationship. One bacterium was phagocytosed and specialized in producing energy through oxidative phosphorylation (OXPHOS), eventually becoming a mitochondrion. Over time, mitochondria have evolved into adaptable organelles focused on energy production, while also acquiring apoptotic functions. Increasing evidence suggests that their role in apoptosis is closely related to nutrient availability and respiratory efficiency [17].

In the central nervous system, mitochondria are abundant within neuronal dendrites and synaptic terminals due to the elevated energy requirements in the brain and the inability to store it [18]. Mitochondria are essential for regulating neuronal activity, both short- and long-term neuronal plasticity, cellular resilience, and behavioral adaptations [19,20,21]. To match neuronal energy needs, mitochondria constantly move along microtube networks, changing their trafficking, distribution, anchoring, and membrane dynamics [22]. Maintaining a balance between energy supply and demand and preserving mitochondrial health is critical for cellular homeostasis and ensuring proper neuronal function [16].

Their main function is energy production in the form of adenosine triphosphate (ATP) through the oxidative metabolism of nutrients. To achieve energy production, firstly, NADH and FADH2 are produced during glycolysis, and the Krebs cycle of β-oxidation of fatty acids is oxidized via the electron transport chain (ETC). Later, ATP is produced by OXPHOS [23].

Mitochondria contain their own DNA, named mitochondrial DNA (mtDNA), which contains 37 genes that encode 13 proteins, which are subunits of the electron transport chain (ETC), 22 tRNA, and two rRNAs. Nuclear DNA (nDNA) codes for the rest of the mitochondrial proteins [24]. Each mitochondrion contains 800 to 1000 copies of mtDNA, which are maternally inherited [23]. In contrast to nDNA, mtDNA is vulnerable to secondary DNA damage due to constant exposure to ROS [25].

Mitochondria contain two specialized membranes, the mitochondrial outer membrane (MOM) and the inner membrane (MIM), and two different compartments, the mitochondrial matrix and intermembrane space [17]. The mitochondrial matrix contains different enzymes that participate in the Krebs cycle and are responsible for the generation of NADH and FADH2 [26], which act as electron donors for the generation of ATP through OXPHOS via the ETC [27,28,29]. The MOM and the intermembrane space are more permeable than the MIM, which contains enzymes involved in the process of ETC and ATP generation via OXPHOS.

### 2.2. Oxidative Phosphorylation (OXPHOS)

**Figure 1:**
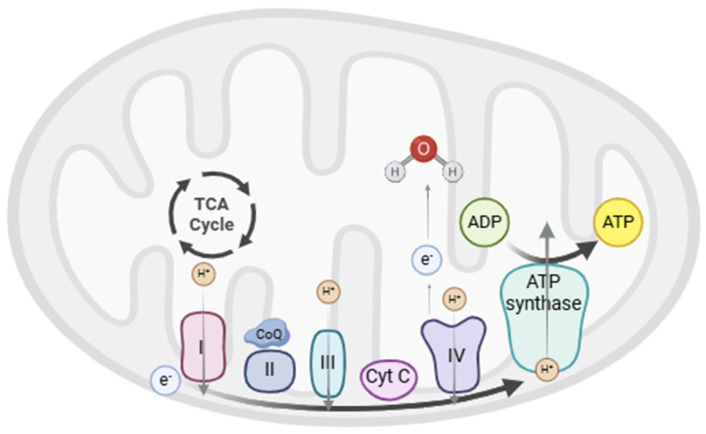
The figure shows the structure of the electron transport chain (ETC) in the internal mitochondrial membrane, which results in ATP production. TCA cycle, tricarboxylic acid cycle; CoQ, coenzyme Q; Cyt C, cytochrome C.

The ETC is found within the MIM [27,28,29] and is composed of five multimeric protein complexes (I–IV and ATP synthase or complex V). These complexes are responsible for ATP production by OXPHOS, in which the proteins of the ETC remove electrons from NADH and FADH2 generated in the previous steps and shuttle them along with the first four complexes. This electron transfer induces the transport of protons from the mitochondrial matrix into the intermembrane space through sequential redox reactions that finally reduce O_2_ to H_2_O in complex IV [23]. Finally, complex V uses the energy accumulated in the proton gradient to phosphorylate adenosine diphosphate (ADP) into ATP [30]. The ETC function is shown in Figure 1.

### 2.3. Oxidative Stress

Since OXPHOS is an imperfect process, when an electrochemical proton gradient is generated for ATP production, electrons can escape and produce a single-electron reduction of O2, forming superoxides and other ROS [31,32]. ROS are highly reactive oxygen-containing free radical molecules that interact with and damage cellular components, including mtDNA, mitochondrial proteins and enzymes, lipids, and membranes, which impair ATP generation and other essential mitochondrial functions [23]. OXPHOS also results in the generation of nitric oxide (NO) and reactive nitrogen species (RNS), which affect cellular proteins. To ameliorate the effects of oxidative damage, cells use different mechanisms to diminish the generation of free radicals or scavenge free radicals using antioxidants [23].

Antioxidant enzymes are key cellular defense mechanisms that reduce oxidative stress and include endogenous molecules, such as superoxide dismutase (SOD), catalase (CAT), glutathione reductase (GR), and glutathione peroxidase (GPx). Other non-enzymatic defenses that protect cells against oxidative stress include vitamins E and C, glutathione (GSH), various carotenoids, and flavonoids [16]. When antioxidant defense is insufficient, oxidative damage in mitochondria is produced [33], which affects ETC function and leads to decreased ATP production, mitochondrial dysfunction, reduced mitochondrial biogenesis, and pathological conditions such as aging, metabolic diseases, and neurodegenerative disorders [34,35,36]. In the last decades, some of the literature has focused on the implications of mitochondria in different pathological processes, including BD [23], where oxidative stress leads to neuronal dysfunction and neurodegeneration, both of which are implicated in BD progression.

### 2.4. Other Functions of Mitochondria

Cellular mechanisms to neutralize ROS include antioxidant defenses and mitochondrial functions aimed at maintaining cellular homeostasis, such as mitochondrial dynamics, biogenesis, and mitophagy [37].

Mitochondria are key organelles involved in calcium homeostasis, one of the apoptosis-triggering factors [38,39]. Both mitochondria and the endoplasmic reticulum serve as major reservoirs of intracellular calcium, avoiding high cytosolic levels that could be toxic to the cell [40]. Mitochondria form signaling hubs with the endoplasmic reticulum through mitochondria-associated membranes (MAMs), which allow rapid transmission of calcium signals and the regulation of lipid synthesis [41]. When cytosolic calcium levels or ATP demands are high, mitochondrial calcium concentrations increase, whereas they decrease when cytosolic levels are low or when the ATP/ADP ratio is high [40]. Calcium modulates OXPHOS in different ways, including direct binding, enhancing post-transcriptional modification, and by the activity of a calcium-dependent binding protein, and can also affect mitochondrial membrane depolarization [42]. It can contribute to faster activity of ETC enzymes and higher ATP production, as well as increased antioxidant defenses [42]. Calcium is also a secondary messenger involved in the regulation of neurotransmission and neuroplasticity in the brain [24]. However, when calcium levels are excessive in the intracellular space or mitochondria, they induce stress and excitotoxicity, ATP synthesis is reduced [43,44], and calcium is ejected through the Na^+^/Ca^2+^ exchanger and mitochondrial permeability transition pore (mPTP) [24]. At the same time, calcium homeostasis is regulated by different proteins, enzymes, and cellular signaling networks [16,45]. Thus, neuronal hyperexcitability related to impaired mitochondrial calcium buffering could contribute to manic episodes and dysfunction in neurotransmitter release, leading to depressive symptoms.

Mitochondria are also implicated in regulating the process of apoptosis through both intrinsic and extrinsic pathways, which allows the brain to remove neurons and glia that are functionally impaired or unable to form neuronal synapses [46]. In the intrinsic mitochondrial-mediated pathway, cellular stress signals, such as high levels of intracellular Ca^2+^, ROS, or the activation of proapoptotic proteins (i.e., Bcl-2 family members) in the MOM [47], trigger a cascade of processes that activate caspases, which results in the cleavage of several proteins, DNA fragmentation, and cell death [48,49]. In the extrinsic pathway, activation of cell surface death receptors enhances processes that alter membrane permeability, resulting in the leakage of proapoptotic factors and apoptosis [17,48]. Apoptosis can contribute to neurodegeneration, particularly in regions associated with mood regulation, such as the prefrontal cortex and hippocampus, and cognitive deficits in BD. Dysregulation in the process of mitochondrial autophagy (mitophagy), a cellular defense mechanism to prevent apoptosis, results in the accumulation of damaged mitochondria, decreased energy production, and increased oxidative stress, which are especially harmful to neurons [50].

Fatty acids are oxidized in the mitochondrial matrix to acyl-CoA through a process called β-oxidation, which allows the obtention of NADH and FADH2, necessary for OXPHOS. The acetyl-CoA resulting from this process can enter the TCA cycle and be oxidized, coupled with the production of reducing power [51].

Mitochondrial activity depends not only on the quantity of these organelles, but also on their morphology and mitochondrial dynamics [52,53,54]. High-energy requirements activate mitochondrial biogenesis. Mitochondria are permanently fusing and fissioning with each other, and damaged mitochondria are rapidly eliminated via mitophagy, which allows mitochondrial networks to meet metabolic demands [37,52]. The loss of fusion and fission abilities results in altered mitochondrial populations [55], which increases individual vulnerability to mood dysregulation.

Mitochondria are also involved in synaptic plasticity, which is related to the effects of glutamate and BDNF. The latter factor increases glucose transport, upregulates mitochondrial biogenesis, and enhances ETC efficiency [56,57]. ATP is required for the mobilization of synaptic vesicles to the active sites of synapses in neurons [58,59]. Mitochondria also play a key role in neurogenesis, the process of neural stem cell proliferation, and differentiation into new neurons through the mitochondrial genome and specific proteins [16].

As reported above, mitochondria meet different complex functions that are closely related, affecting one another, which explains why several mitochondrial roles are impaired in pathological conditions.

## 3. Mitochondrial Dysfunction in Bipolar Disorder

Despite being the organ with the highest energy consumption, the brain is unable to store glycogen and depends on mitochondrial function for energy production, which explains the large number of mitochondria and the high amount of ATP produced in this organ. However, ROS and RNS production are also abundant in the brain, which is vulnerable to oxidative damage and potential impairments in mitochondrial function [18].

In BD, mitochondrial dysfunction and impaired energy metabolism have been reported in different states of the illness, including euthymia [10,11], potentially impacting the severity and course of the illness [12,13]. Some of the observed changes associated with mitochondrial function include altered mitochondrial-related gene expression, impaired mitochondrial biogenesis, structural and dynamic abnormalities, fluctuations in OXPHOS, and altered metabolite levels [60]. Genetic and environmental differences associated with race, as well as physiological changes related to aging, can modify mitochondrial function and potentially alter the clinical course, severity, and response to treatment in BD [21]. Increasing age is associated with a lower efficiency of mitochondrial OXPHOS, decreased ATP production, and increased ROS generation. In BD, aging can exacerbate these deficits [21].

**Figure 2:**
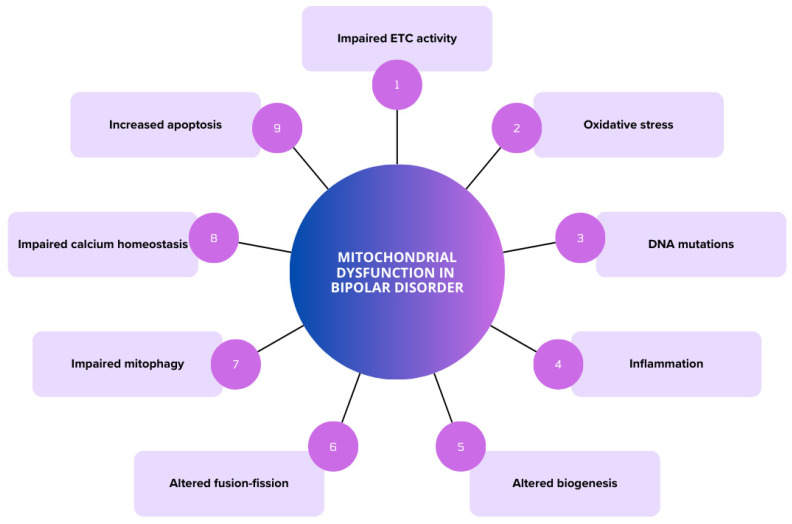
Altered mitochondrial functions in bipolar disorder. ETC: electron transport chain.

Some evidence supports the hypothesis regarding the contribution of mitochondrial dysfunction in BD, particularly in mood and cognition [14,15], with specific variations according to mood state [11]. For instance, in manic states, energy production and oxidative stress are increased, firstly supported by the presence of antioxidative defenses. However, eventually, the defensive pathways become overwhelmed and mitochondrial function deteriorates, leading to cellular damage [11]. The cumulative evidence points to greater mitochondrial impairments in BD type I compared to BD type II, since the former involves greater fluctuations in energy production due to the presence of manic episodes characterized by increased metabolic demand. In addition, higher levels of oxidative stress markers have been observed in BD type I. Calcium dysregulation has been suggested to correlate with manic symptoms, and increased mitochondrial biogenesis and apoptosis have been described in manic episodes [16]. Despite this, the available evidence shows that mitochondrial dysfunction is present in all phases of the illness, not only with altered processes but also with abnormalities in the structure and distribution of mitochondria within the cell, despite growing evidence pointing to specific differences according to mood states. Figure 2 illustrates the current evidence of mitochondrial dysfunction in BD.

### 3.1. Metabolic Changes

Evidence from neuroimaging studies and analysis of postmortem brain tissue from individuals with BD revealed a decreased number of neuronal and glial cells and reduced brain volume in the prefrontal and limbic areas [61,62]. These findings are associated with a reduction in oxidative bioenergetic production, favoring anaerobic glycolysis, and subsequently impaired neuroplasticity, phospholipid metabolism, and calcium regulation. Moreover, changes in neurometabolite levels, including high-energy compounds, have been observed in patients with mood disorder, such as lower levels of phosphocreatine (PCr), which serves as the cell’s energy storage. Changes in N-acetyl-aspartate (NAA), ADP, and ATP levels have also been reported in BD and are associated with mitochondrial dysfunction [61,62].

Some studies have identified enzymatic abnormalities in creatine kinase (CK), which catalyzes the production of ATP from ADP and PCr in cases of high-energy demand, and downregulation of CK in BD [63,64]. Specifically, CK in the hippocampus and dorsolateral prefrontal cortex was downregulated in individuals with BD, which could explain the reduction in high-energy phosphates previously observed in this population. A significant reduction in the CK forward reaction rate constant was also found in BD [63]. These results support previous findings indicating that BD patients are able to maintain average brain concentrations of high-energy compounds at rest but exhibit underlying abnormalities in situations of high-energy demand [63]. An increase in lactate levels and decreased brain intracellular pH have also been described, which suggests a tendency to shift from OXPHOS to glycolysis for energy obtention in BD. Increased lactate in manic phases, which has been observed in various brain regions (frontal cortex, caudate, and cingulate cortex), suggests either heightened ATP demand or defective oxidative metabolism [11]. Adolescent individuals with BD, compared to healthy controls, presented increased lactate levels, which were positively correlated with cell-free mtDNA [65].

A review aimed at summarizing data from proteomic analyses of postmortem brains collected from patients with BD identified 95 proteins as altered, most of them related to the TCA cycle and the ETC, and others with antioxidant enzymes, which might contribute to a better understanding of the impaired metabolic mechanisms in BD [66].

### 3.2. Electron Transport Chain (ETC)

#### 3.2.1. Enzymatic Activity

Results from studies examining postmortem brain tissue, skeletal muscle, or blood samples have shown alterations in mitochondrial enzymatic activities, some of which are associated with the TCA cycle and ETC, in patients with BD, especially during stress conditions. Reduced expression and activity of ETC complexes and decreased activity of citrate synthase, which is involved in the TCA cycle, have been reported [60,67,68]. Different mitochondria-related genes have been found to be downregulated in BD compared to controls [69].

#### 3.2.2. Mitochondrial Respiratory Capacity

Recent evidence has focused on the assessment of interindividual variability to explore differences in mitochondrial function between different mood states in BD, based on the hypothesis of a biphasic pattern of energy availability in this illness. Mitochondrial respiratory capacity was assessed in PBMCs of patients with BD admitted to an acute psychiatric ward during an acute mood episode and after clinical remission [70]. The mitochondrial oxygen consumption capacity was lower in patients with BD during a manic or depressive episode compared to those in clinical remission, and bipolar depression was associated with lower levels of mitochondrial respiratory capacity compared to mania [70]. In addition, specific clinical features, including energy, motor activity, insomnia, and language/thought disorder, have been shown to be correlated with mitochondrial respiratory capacity [70].

A subsample from a previous study was selected to determine the correlation between mitochondrial respiration analyses and aerobic capacity during exercise effort tests on a cycle ergometer and the differences between different mood states [71]. When comparing acute affective episodes with clinical remission, no significant differences in aerobic capacity were observed during the effort test. In euthymia, pre-exercise oxygen uptake tended to negatively correlate with maximal mitochondrial oxygen consumption capacity, and maximal oxygen uptake during exercise was inversely correlated with basal mitochondrial respiration, suggesting an association between mitochondrial ETC dysfunction and impaired aerobic respiration, which could be a risk factor for increased anaerobic respiration and oxidative stress [71].

Compared to healthy controls, patients with BD have shown a decrease in the mitochondrial health index (MHI), which includes mitochondrial functional capacity in human leukocytes, considering respiratory chain enzymatic activities and mtDNA copy number (mtDNAcn) [72]. This has been correlated with plasma circulating cell-free mtDNA [73]. 

### 3.3. Oxidative Stress

An increase in ROS production and reduced antioxidant capacity have been reported in BD [10], with increased lipid peroxidation products in the cingulate cortex [10] and increased markers of oxidative and nitrosative damage in the prefrontal cortex [74,75]. Results from a meta-analysis assessing oxidative stress markers in individuals with BD showed an increase in markers of lipid peroxidation, DNA/RNA damage, and nitric oxide [76].

Studies in the postmortem brains of individuals with BD have shown lower expression of SOD, microsomal glutathione S-transferase, and GPx in frontal areas, lower expression of GPx in the hippocampus [77,78], and reduced activity of SOD and catalase [79,80]. Some evidence shows increased SOD activity during manic and depressive episodes [79,81,82]; while other authors reported decreased SOD levels during manic episodes [83]. Results in euthymic patients are controverted [79,84]. Increased GPx activity of GPx been reported in euthymic BD patients but not in acute mood episodes [85]. However, other authors found increased GPx levels in patients with bipolar depression compared to healthy controls [86], and others did not find significant differences between healthy controls and acute mood episodes [75,79]. Elevated catalase levels have been reported in bipolar depression [86]. In addition, compared to healthy controls, decreased GSH levels were observed among patients in the late stages of BD [75]. 

Sex differences in mitochondrial function in BD have been associated with differences in oxidative stress. Estrogens have been shown to enhance mitochondrial efficiency by increasing ATP production, improving antioxidant defenses, and regulating mitochondrial biogenesis. These effects may help explain the lower neuronal damage, higher prevalence of depressive episodes in women than in men, and greater functional impairment in men during manic episodes [45].

### 3.4. Calcium Homeostasis

Calcium uptake in the mitochondrion regulates intracellular calcium signaling, controls the rate of energy production, induces cellular death, and controls neuronal excitability [87]. Altered intracellular calcium levels are a consistent finding in patients with BD. Studies performed on the brains of individuals with BD have shown increased calcium levels during manic states [45,88], as well as gene expression changes in calcium-related signaling pathways. Changes in PKA/PKC signaling in BD neurons have been found, indicating that these pathways might be related to neuronal hyperexcitability [61]. Mutations affecting Calcium/Calmodulin Dependent Protein Kinase Kinase 2 (CaMKK2), which plays a main role in neuronal calcium-calmodulin signaling, leading to reduced CaMKK2 activity and decreased BDNF expression [89,90], have been associated with BD [91].

Calcium signaling also regulates cell death through crosstalk between the endoplasmic reticulum and mitochondria. Calcium overload in mitochondria induces endoplasmic reticulum stress, calcium leakage, collapse of the mitochondrial membrane potential, termination of OXPHOS, osmotic changes, mitochondrial swelling, inner membrane remodeling, and opening of the mitochondrial permeability transition pore (mPTP) [87]. It culminates in MOM permeabilization and cytochrome c release, which induces apoptosis [61].

Excess calcium affects both neuronal excitability and signaling cascades that regulate gene expression, disrupting neuronal processes such as dendrite development, synaptic plasticity, and excitatory/inhibitory balance [92]. These changes might be related to disturbances in the homeostatic control of cellular physiology.

### 3.5. Mitochondrial Morphology and Dynamics

Altered mitochondrial morphology, distribution, and degradation have been reported in patients with BD. In both prefrontal neurons from postmortem brain samples and peripheral cells obtained from individuals with BD, a larger number of smaller-sized mitochondria have been found [93,94]. Mitochondria marginalization in the intracellular distribution in peripheral cells was observed, along with atypically shaped mitochondria [94]. Moreover, in iPSC-derived hippocampal dentate gyrus-like neurons from patients with BD [94], smaller mitochondria were found compared to controls, along with altered fusion and fission processes, and altered mitochondrial size [94]. These findings were observed in patients with euthymia. Supporting these findings, a downregulation of the mitochondrial fusion-related proteins Mfn-2 and Opa-1 and an upregulation of the fission protein Fis-1 were observed in PBMCs from patients with BD [95], suggesting that the imbalance in mitochondrial dynamics might explain the abnormal mitochondrial morphology observed in these patients, which could increase the vulnerability of these individuals to present bioenergetic alterations in acute episodes of the illness. In BD, MHI was negatively correlated with Fis-1 levels and positively correlated with Opa-1 and LC3 levels. In addition, cell-free mtDNA was negatively correlated with Opa-1 and LC3 and positively correlated with Fis-1. Subjects with longer illness duration, higher depressive symptom scores, and worse functional status had lower MHI and higher cell-free mtDNA [73]. 

### 3.6. Mitochondrial Degradation and Apoptosis

In BD, PBMCs display downregulation of mitophagy-related proteins, altered mitochondrial fission and fusion processes favoring fission, and increased caspase-3 levels [95,96]. This indicates that the number of damaged mitochondria may exceed the capacity of mitophagy, causing apoptosis to become the dominant pathway for minimizing tissue damage [97,98]. Indeed, in the hippocampus of BD patients, upregulation of proapoptotic genes, such as FAS, BAK, and APAF-1 [78], and downregulation of the antiapoptotic protein Bcl-2 due to various polymorphisms have been observed [97].

Moreover, the PI3K/Akt pathway, which activates mTOR and stimulates OXPHOS [11], is upregulated in mania and is activated by oxidative stress and IL-6 [78]. Akt promotes mitochondrial survival by inhibiting cytochrome c release into the cytosol, which is the last step in mitochondrial apoptosis [99].

Chronic oxidative stress, as in BD, activates GSK-3α and GSK-3β, with greater activation in mania than in depression. Their inhibition correlates with clinical improvement [11]. GSK-3 activation promotes cellular apoptosis and is enhanced by TNF-α, which paradoxically has a neuroprotective role. In mania, TNF-α activation of GSK-3 promotes neuronal survival by upregulating NFκβ, which inhibits TNF-α mediated apoptosis, inhibits OXPHOS, and promotes aerobic glycolysis. In addition, TNF-α inhibits mitochondrial biogenesis, which is compensated by the increased activity of SIRT-1 [11]. Through insulin signaling, SIRT-1 is involved in the regulation of glucose and lipid metabolism and protects cells against inflammation and oxidative stress. It activates PGC1α, thereby promoting glucose uptake and mitochondrial biogenesis [93]. In comparison with bipolar depression and healthy controls, BD patients with manic episodes have shown increased levels of NFκβ and SIRT-1 [11]. SIRT-1 levels are decreased in bipolar depression compared to euthymia, and TNF-α levels seem to be lower in depression than in mania [11]. NFκβ also causes elevated cytoplasmic CREB levels in BD, which is highly relevant given CREB’s role in mediating BDNF’s antioxidative effects, with lower levels observed in mania compared to depression and in patients with BD compared to controls [100]. Additionally, CREB involvement in neurogenesis has been found to be diminished in depression [101].

### 3.7. Inflammation

As observed in other mental disorders, a proinflammatory environment has been described in all phases of BD, with an increase in cytokine levels, especially IL-1β, IL-6, and TNF-α, and increased nitric oxide in the brain and plasma. These changes have been found to be greatest in mania and also higher in bipolar depression compared to unipolar depression [18].

### 3.8. Genetics

Studies focusing on genetic evidence also support the involvement of mitochondrial dysfunction in BD [101]. Genome-wide association studies (GWASs) have identified multiple loci associated with BD susceptibility, including CACNA1C, ANK3, ODZ4, SYNE1, and TRANK1 [102,103], albeit with relatively modest effect sizes. The potential involvement of de novo protein-altering mutations and calcium-related genes has been associated with BD pathogenesis and early disease onset [104,105]. Recent evidence identified genetic variants in BD, suggesting that mitochondrial variants in genes related to NADH dehydrogenase may contribute to the pathogenesis of BD via dysfunction of energy production [106]. While no direct mutations in mtDNA have been linked to BD, specific mtDNA variants, such as a rare gene variant, 3644T>C, appear to be more prevalent in patients with BD compared to healthy controls [107,108]. This causes a decrease in mitochondrial membrane potential and complex I activity by amino acid substitution in NADH-ubiquinone dehydrogenase subunit I (ND1). 

Genetic variations in the purinergic system and clock genes have also been reported in BD [11]. Mitochondrial OXPHOS, redox states, and mitochondrial dynamics are regulated in a circadian manner, and daily rhythms in cytochrome c oxidase activity, mitochondrial membrane potential, and calcium release from the mitochondria have been found [109]. The transcriptional coactivator PGC-1α, the main regulator of mitochondrial biogenesis and energy metabolism, plays an important role in the circadian clock, which is key to maintaining cellular health [109]. Polymorphisms in clock genes, which can modify cellular sensitivity to oxidative stress, have been associated with a higher risk of developing severe forms of BD. These genes control circadian NAD+ concentrations, which increase SIRT-1 and SIRT-3 activities, and this stimulates OXPHOS. Both NAD+ and SIRT-1 directly activate ATP synthesis and upregulate circadian genes, suggesting an influence of this pathway in affective disorders [18].

### 3.9. Mitochondrial DNA

Studies on postmortem brain samples of mtDNA show controverted findings. Some of them display an increased prevalence of mtDNA deletions [110,111], whereas others do not replicate these results [112]. Regarding mtDNAcn, studies on BD exhibit variability, with some showing reduced copy numbers, particularly during mood episodes [113]. Decreased mtDNAcn and accelerated epigenetic aging in the hippocampus of patients with BD were described in a meta-analysis [114]. In addition, fluctuations in mtDNAcn are correlated with the severity of depressive and manic symptoms [115]. Recent evidence showed no differences in mtDNAcn between patients with BD and healthy controls in whole blood [116], and others displayed reduced mtDNAcn in plasma [117]. Higher cell-free mtDNA plasma levels have been described in BD and are correlated with mitochondrial health. Patients with higher cell-free mtDNA levels experience longer illness durations, deteriorated functionality, and more intense depressive symptoms, suggesting that this might serve as a marker of symptom severity [73].

### 3.10. Other Changes in Mitochondrial Function

In BD, the purinergic system appears dysregulated [118] and involves increased activity in oxidative stress-related pathways like SIRT-1, AMPK, PKA, PKC, GSK, and inositol triphosphate, as well as increased levels of antiapoptotic proteins, such as Bcl-2, PI3K, mTOR, Akt, and uric acid. Their activation drives OXPHOS, which leads to increased oxidative stress. Uric acid levels seem to be increased in all phases of BD, particularly in mania, and they facilitate greater mitochondrial function by enhancing calcium uptake, increasing membrane potential, and ATP production [119]. Uric acid also scavenges peroxynitrite, which has high mitotoxic activity [18], and other neuroprotective effects, including adaptive responses to oxidative stress.

Cytochrome c oxidase, the terminal respiratory enzyme, is a metabolic marker of neuronal functional activity; depressive symptoms have been associated with its alterations [120].

Depression has been correlated with hypothalamic-pituitary-adrenal (HPA) axis hyperactivity, elevated glucocorticoid levels, and increased mitochondrial activity [16,18]. Glucocorticoids inhibit apoptosis by forming a complex with the antiapoptotic protein Bcl-2 to inhibit the formation of Bax-containing pores on the MOM. They also reduce the release of calcium and cytochrome c from the mitochondria, which inhibits apoptosis [101]. However, chronic elevation causes neuronal toxicity, ETC dysfunction, excessive ROS generation, apoptosis, and cell death [121].

Regarding neurotransmitters, glutamate dysregulation has been associated with mood disorders, with elevated levels observed during acute episodes. Oxidative and nitrosative stresses have been associated with higher dopamine transmission and impaired dopamine transporter (DAT) function in mania [122]. However, dopamine and uric acid levels act in a synergistic way to repair oxidative damage [11]. Dopamine can protect neurons against glutamate-induced excitotoxicity and confer antiapoptotic effects. Thus, high dopamine and glutamate levels together with high uric acid levels may not be responsible for the expected detrimental effects; moreover, pro-apoptotic signals may induce the expression of antiapoptotic genes and stimulate OXPHOS [11].

## 4. Potential Mitochondrial-Related Therapies in Bipolar Disorder

Despite the demonstrated efficacy of current therapies for BD, its treatment remains challenging due to non-responder rates. Early intervention seems to be the key to preventing and treating mitochondrial dysfunction and delaying neuroprogression. Thus, there is an urgent need to identify novel targets that may lead to improved efficacy and prevent the relapse of this disease. Different agents have been shown to modulate mitochondrial activity and have potential effects on mood states in BD. For instance, photobiomodulation has been reported to specifically target mitochondrial pathways and has shown promise in BD through amelioration of residual affective symptoms [109]. In addition, psychotherapy has been postulated as a therapeutic strategy to reduce ROS levels and systemic inflammation by improving stress resilience. Its capacity to reduce HPA dysregulation, inflammation, and oxidative damage highlights its potential role in improving mitochondrial function in BD. Psychotherapy also promotes neuroplasticity by supporting healthy neuronal connections and indirectly improving mitochondrial function. Psychotherapies, such as cognitive-behavioral therapy, mindfulness-based interventions, and interpersonal and social rhythm therapy, have been suggested to be beneficial for mitochondrial health. Behavioral changes encouraged by psychotherapy, such as regular physical activity, improved sleep, and balanced nutrition, further protect and optimize mitochondrial function. However, further evidence is still needed. Thus, combining psychotherapy with pharmacological and lifestyle interventions may provide comprehensive benefits for mitochondrial health and mood stabilization [121]. Other therapeutic agents associated with mitochondrial functions are reported below.

### 4.1. Pharmacological Treatments

According to current guidelines, different drugs, such as mood stabilizers and antipsychotics, have been shown to be effective in the treatment of different phases of BD, including acute mania, mixed episodes, depressive episodes, and maintenance treatment [3]. However, the precise beneficial mechanisms of BD remain unclear. They have been postulated to enhance energy metabolism [122] and have been shown to affect different mitochondrial-related functions. Evidence from in vitro studies suggests that lithium might stabilize mitochondrial membrane potential, reduce DNA damaging effects, avoid calcium-induced apoptosis by antagonizing the mPTP, and confer antiapoptotic properties [122]. Other evidence has shown decreased levels of DNA methylation in BD, increased levels of glutathione transferase [123,124], reduced apoptosis, increased catalase activity [125], and enhanced activity of complex I in the prefrontal cortex [126] and complexes II and III in the human frontal cortex [127].

Valproate and lithium have been found to increase the expression of Bcl-2, which leads to the inhibition of proapoptotic enzymes, such as caspase 3 [122]. They are also associated with the inhibition of GSK-3 enzyme activity, which modulates the gene expression of proteins involved in apoptosis, synaptic plasticity, and cellular resilience [128,129]. Lithium and valproate also increase BDNF production, decrease glutamate-induced excitotoxicity, and inhibit NMDA receptor-mediated calcium influx [122]. Valproate also affects mitochondrial epigenetics by the inhibition of histone deacetylase [130].

### 4.2. Nutrient Therapies

Considering that mitochondria regulate energy production, regulate calcium and apoptotic processes, and are central to facilitating neuronal plasticity, dysfunctional mitochondria and oxidative stress can result in neuronal damage and in the development and progression of BD. Different studies have assessed the efficacy of mitochondrial modulators as potential adjunctive treatments in BD [131], including dietary supplements or nutraceuticals, which have been shown to enhance mitochondrial function and brain energy metabolism, mainly by the reduction of oxidative stress.

Some of the studied agents include N-acetyl-cysteine (NAC), acetyl-L-carnitine (ALCAR), S-adenosylmethionine (SAMe), coenzyme Q10 (CoQ10), alpha-lipoic acid (ALA), creatine monohydrate (CM), vitamin D, and melatonin [132,133]. Their potential benefits in the treatment of BD remain unclear. Different meta-analyses have reported the benefits of NAC in mood disorders [134,135], but evidence regarding other mitochondrial regulators remains controversial. A recent meta-analysis of randomized clinical trials found a moderate antidepressant effect of mitochondrial modulators overall. When they were assessed individually, only NAC and CoQ10 showed significant differences compared to placebo [131]. Regarding manic symptoms, overall, mitochondrial modulators did not show statistical differences from placebo [131]. However, a larger number of studies with long-term assessments are needed to determine whether mitochondrial modulators can serve as effective treatments in patients with BD.

The World Federation of Societies of Biological Psychiatry (WFSBP) and Canadian Network for Mood and Anxiety Treatments (CANMAT) Taskforce weakly support the use of omega-3 for bipolar depression. Adjunctive eicosapentaenoic acid (EPA) and N- acetylcysteine are also recommended as third-line treatment options to use adjunctively to other medications in bipolar depression. Whereas different phytoceuticals are recommended for unipolar depression, including St John’s wort, saffron, curcumin, and lavender, they have not been recommended for bipolar depression [3]. 

Consumption of nutrients is also key to maintaining brain health and its functioning, and diet can affect mitochondrial activity, inflammation, oxidative stress, and neuroprogression [136,137,138]. Oxidants are linked to membrane-related pathology in the brain [121]. Consequently, antioxidant agents may alleviate affective symptoms and should be explored as adjunctive therapies, especially in those patients exhibiting immune dysregulation [121].

The effects of specific diets on mood disorders are still not clear despite evidence reporting that they can alter several biological processes and act as mood stabilizers, since randomized clinical trials in BD are still lacking. The ketogenic diet consists of a low-carbohydrate diet that changes the energy source of the organism by switching from glucose to ketone bodies [139]. Beneficial effects of the ketogenic diet have been suggested in BD because it could be associated with an increase in blood acidity, elevation in GABA levels, regulation of GABA type A receptors, and inhibition of α-amino-3-hydroxy-5-methyl-4-isoxazolepropionic acid (AMPA) receptors by medium-chain fatty acids [139]. Thus, a ketogenic diet affects glutamate metabolism and nerve cell metabolism through the use of ketone bodies as energy sources [139]. It upregulates mitochondrial antioxidant activity and prevents mtDNA oxidant-induced damage [139].

### 4.3. Physical Therapies

Finally, physical exercise has been suggested as an effective therapy not only to improve physical well-being but also to enhance mitochondrial activity in BD. Lifestyle management, including attention to diet, substance use, smoking, and physical activity, is recommended alongside any psychological or pharmacological intervention for BD [3]. Exercise has been shown to contribute to adult neurogenesis and improve mitochondrial functions via its multiple biological effects, such as the production of BDNF, neuroplasticity, and the rate of apoptosis in the hippocampus [140], as well as regulation of mitochondrial ETC function, promotion of fission, and mitochondrial biogenesis [141,142]. Exercise can also attenuate the inflammatory response, which negatively affects adult neurogenesis [142,143]. However, limited evidence has shown the ability of exercise to mediate mitochondrial functions in neural tissues [141]. Although evidence on BD is still lacking, current guidelines support physical exercise as an adjunctive treatment for major depressive disorder [3].

## 5. Conclusions

Mitochondria are cellular organelles involved in different functions and are responsible for cellular energy production in the form of ATP. Current evidence suggests the implication of mitochondria in the pathophysiology of psychiatric disorders, especially BD, where altered mitochondrial function has been described in all phases. This highlights mitochondrial dysfunction, especially energy production and oxidative stress, as potential therapeutic targets for the treatment of BD. The limitations of this review include the non-systematic methodology, variety of mitochondrial-related functions associated with BD, heterogeneous study designs and evidence base, preliminary evidence of some of the findings described, and limited recommendations regarding the use of mitochondrial modulators in BD. Future evidence will allow the identification of specific mitochondria-related biomarkers and the development of new therapies focused on mitochondrial functions that might be used as the main or adjunctive strategies. Future research should determine the specific effects of these therapies on energy production, oxidative stress, and other mitochondrial-related functions.
